# Dibucaine Mitigates Spreading Depolarization in Human Neocortical Slices and Prevents Acute Dendritic Injury in the Ischemic Rodent Neocortex

**DOI:** 10.1371/journal.pone.0022351

**Published:** 2011-07-15

**Authors:** W. Christopher Risher, Mark R. Lee, Ioulia V. Fomitcheva, David C. Hess, Sergei A. Kirov

**Affiliations:** 1 Graduate Program in Neuroscience, Georgia Health Sciences University, Augusta, Georgia, United States of America; 2 Department of Neurosurgery, Georgia Health Sciences University, Augusta, Georgia, United States of America; 3 Department of Neurology, Georgia Health Sciences University, Augusta, Georgia, United States of America; 4 Brain and Behavior Discovery Institute, Georgia Health Sciences University, Augusta, Georgia, United States of America; National Institute of Health, United States of America

## Abstract

**Background:**

Spreading depolarizations that occur in patients with malignant stroke, subarachnoid/intracranial hemorrhage, and traumatic brain injury are known to facilitate neuronal damage in metabolically compromised brain tissue. The dramatic failure of brain ion homeostasis caused by propagating spreading depolarizations results in neuronal and astroglial swelling. In essence, swelling is the initial response and a sign of the acute neuronal injury that follows if energy deprivation is maintained. Choosing spreading depolarizations as a target for therapeutic intervention, we have used human brain slices and *in vivo* real-time two-photon laser scanning microscopy in the mouse neocortex to study potentially useful therapeutics against spreading depolarization-induced injury.

**Methodology/Principal Findings:**

We have shown that anoxic or terminal depolarization, a spreading depolarization wave ignited in the ischemic core where neurons cannot repolarize, can be evoked in human slices from pediatric brains during simulated ischemia induced by oxygen/glucose deprivation or by exposure to ouabain. Changes in light transmittance (LT) tracked terminal depolarization in time and space. Though spreading depolarizations are notoriously difficult to block, terminal depolarization onset was delayed by dibucaine, a local amide anesthetic and sodium channel blocker. Remarkably, the occurrence of ouabain-induced terminal depolarization was delayed at a concentration of 1 µM that preserves synaptic function. Moreover, *in vivo* two-photon imaging in the penumbra revealed that, though spreading depolarizations did still occur, spreading depolarization-induced dendritic injury was inhibited by dibucaine administered intravenously at 2.5 mg/kg in a mouse stroke model.

**Conclusions/Significance:**

Dibucaine mitigated the effects of spreading depolarization at a concentration that could be well-tolerated therapeutically. Hence, dibucaine is a promising candidate to protect the brain from ischemic injury with an approach that does not rely on the complete abolishment of spreading depolarizations.

## Introduction

Within minutes of focal stroke onset, a spreading depolarization originates from an area of severely decreased blood flow known as the ischemic core [1]–[4]. In the core, where neurons do not repolarize, this prolonged spreading depolarization is called the anoxic or terminal depolarization [4]. It propagates into the ischemic penumbra along a decreasing gradient of metabolic stress and into normoxic tissue where it becomes short-lasting [5]–[10]. Recurring spontaneous spreading depolarizations arising at the perimeter of the core propagate throughout the penumbra for hours to days in animal models and patients [11]–[17]. The prolonged duration of recurring spreading depolarizations further elevates metabolic stress in the penumbra due to the mismatch between energy supply and needs for recovery. Eventually penumbral neurons and astrocytes remain depolarized and overloaded with Ca^2+^, recruiting the tissue into infarct [11], [16], [18]–[20]. It has been proposed that a useful anti-stroke drug should abrogate spreading depolarizations without depressing normal synaptic function [4], [21]. We have recently shown that one such candidate is dibucaine, an FDA-approved local amide anesthetic and sodium channel blocker that potently inhibits terminal depolarization in rat brain slices while preserving synaptic function [22]. However, nearly all clinical trials of drugs that were effective in animal models of stroke/ischemia have failed, with the limited complexity of animal models compared to human stroke as well as fundamental differences between the brains of animals and humans assumed as contributing factors [23]. It is recognized by stroke experts that human brain slices are a crucial intermediate assay to forecast translational success before clinical trials [24]. Towards this end, we used light transmittance (LT) imaging to confirm that dibucaine diminishes the impact of terminal depolarization on live human neocortical slices prepared from pediatric brain tissue mostly resected for the treatment of intractable epilepsy as well as for neoplasm removal. We supplemented human slice experiments with a sophisticated *in vivo* approach using real-time 2-photon laser scanning microscopy (2PLSM) to show that spontaneous spreading depolarization-elicited dendritic damage is greatly reduced following photothrombosis-induced focal ischemia in dibucaine-treated mice. This success of dibucaine in reducing the negative effects of spreading depolarization makes it a strong candidate for further investigation to prevent spreading depolarization-induced neuronal damage in the acutely injured brain.

## Materials and Methods

### Ethics Statement

All protocols involving human tissue were approved by the Institutional Review Board at Georgia Health Sciences University. Written parents' informed consent documents and written children's assent documents were obtained prior to each study. All animal procedures were carried out in strict accordance with the recommendations in the Guide for the Care and Use of Laboratory Animals of the National Institutes of Health. The protocol for this study was specifically approved by the Animal Care and Use Committee at Georgia Health Sciences University (Permit Number 08-05-051). All surgery was performed under urethane anesthesia and all efforts were made to minimize animal discomfort and reduce the number of animals used.

### Human Slice Preparation and Solutions

Human neocortical slices were made as previously described [25] from tissue resected from a total of 23 pediatric patients (average age∼9 y.o.) of both genders undergoing surgery for pharmacoresistant epilepsy (20 patients from whom tissue was used in the described experiments; these patients are listed in the Patient Table S1). Written parents' informed consent documents and written children's assent documents were obtained prior to each study. All protocols were approved by the Institutional Review Board at Georgia Health Sciences University. Coronal slices (400 µm) were cut using a vibrating-blade microtome (VT1000S, Leica Instruments) with ice-cold oxygenated sucrose-based artificial cerebrospinal fluid (Sucrose-ACSF) containing (in mM) 100 sucrose, 60 NaCl, 2.5 KCl, 26 NaHCO_3_, 1.25 NaH_2_PO_4_, 1 CaCl_2_, 5 MgCl_2_, 10 glucose, pH 7.4, 290 mOsm/kg H_2_O. Slices were transferred into an incubation chamber and maintained at room temperature for at least 1 hour in standard oxygenated ACSF containing (in mM) 125 NaCl, 2.5 KCl, 26 NaHCO_3_, 1.25 NaH_2_PO_4_, 2 CaCl_2_, 1 MgCl_2_, 10 glucose, pH 7.4, 290 mOsm/kg H_2_O. Some slices were transferred into a separate incubation chamber and pretreated for 1 hour at room temperature with ACSF containing 1 or 10 µM dibucaine hydrochloride (cinchocaine) [2-butoxy-N-(2-diethylaminoethyl) quinoline-4-carboxamide hydrochloride] or 0.1, 1, 5 or 100 µM minocycline. For imaging, a slice was transferred into a submersion-type imaging/recording chamber (RC-29, Warner Instruments) at 34°C and held down by an anchor (SHD-27LP/2, Warner). For oxygen/glucose deprivation (OGD, 20 min duration), ACSF was bubbled with 95%N_2_–5%CO_2_ and glucose lowered to 1 mM. NaCl was added to ACSF to osmotically balance the removed glucose. 100 µM ouabain was added to ACSF for chemical ischemia (15 min duration). Terminal depolarization induced by ouabain has been shown to elicit changes in LT, electrophysiology and drug effects indistinguishable from those seen following OGD-induced depolarization [21], [22], [26]. Therefore, we use the term ‘terminal depolarization’ for both OGD-induced depolarization and for the ouabain-induced depolarization. All chemicals were from Sigma Chemical unless indicated otherwise.

### Intrinsic Optical Imaging

Intrinsic optical signals were acquired as previously described following standard protocols [22], [27], [28]. Slices were transilluminated with a broadband, halogen light source (Carl Zeiss) through a near-infrared pass filter. LT was collected with 2.5×/0.075NA air objective with dipping cone attachment [29] using the motorized upright Axioscope-2FS microscope (Zeiss) with IR-1000 CCD camera (Dage-MTI). Image frames were acquired at 30 Hz, digitized and averaged using a frame grabber controlled by Scion Imaging Software. Each image was generated by averaging 128 frames. The control image (T_cont_) was subtracted from each subsequent experimental image (T_exp_), resulting in an image series that revealed changes in LT over time [21], [22], [27], [28]. The LT change was expressed as the digital intensity of subtracted images (T_exp_-T_cont_) and displayed using a pseudocolor intensity scale. ‘Zones of interest’ were created in the images to quantify the data, with at least one zone positioned near the origin of the terminal depolarization front to record onset time. The difference (T_exp_-T_cont_) was normalized by dividing by T_cont_ which varies across the slice depending on the zone sampled. To accommodate for potential sample variables, we always compared control and drug-treated slices within tissue from the same patient.

### Transgenic Mice

All animal procedures follow NIH guidelines and underwent yearly review by the Animal Care and Use Committee at Georgia Health Sciences University (Permit Number 08-05-051). All efforts were made to minimize animal discomfort and reduce the number of animals used. Founders of the *B6.Cg-Tg(Thy1-EGFP)MJrs/J* colony [GFP-M] were kindly provided by Dr. J. Sanes (Harvard University, Boston, MA). 12 GFP-M and 12 wild-type *C57BL/6J* male and female mice (average age∼4 months) were used in this study. Control data for *in vivo* experiments were taken from 24 GFP-M male and female mice (average age∼4 months) which were tested without drug treatment in a previous study [15] concurrently with the dibucaine-treated mice.

### Mouse Slice Preparation and Electrophysiology

Acute murine slices (400 µm) were made from *C57BL/6J* mice according to standard protocols [30], [31]. Field excitatory postsynaptic potentials (fEPSPs) were recorded in the middle of *stratum radiatum* of hippocampal area CA1. Signals were recorded with MultiClamp 200B amplifier, filtered at 2 kHz, digitized at 10 kHz with Digidata 1322A interface board and analyzed with pClamp 9 software (Molecular Devices). Stimuli (100 µs duration) ranging from 10–200 µA were applied via small concentric bipolar electrode (25 µm pole separation; FHC). The slope function (mV/ms) of the fEPSP was measured from the steepest 400 µs segment of the negative field potential.

### Preparation of Mice for *In Vivo* Imaging

Craniotomy for the optical window followed standard protocol [15]. Mice were anesthetized with an intraperitoneal injection of urethane (1.5 mg/g body weight) with heart rate monitored (450–650 beats/min) using MouseOx® pulse oximeter (STARR Life Sciences). Depth of anesthesia was assessed by toe pinch and heart rate monitoring, maintained with 10% of the initial urethane dose if necessary. Oxygen saturation level remained >90% for duration of experiments, indicating that mice were respiring properly under our imaging conditions. Hydration was maintained by intraperitoneal injection of 100 µl 0.9% NaCl with 20 mM glucose at 1 hour intervals. A 0.1 ml bolus of 5% (w/v) Texas Red Dextran (70 kDA) (Invitrogen) in 0.9% NaCl was injected into the tail vein for blood flow visualization. An optical chamber was constructed by covering the intact dura with a thin layer of 1.5% agarose prepared in a cortex buffer containing (in mM) 135 NaCl, 5.4 KCl, 1 MgCl_2_, 1.8 CaCl_2_, and 5 HEPES, pH 7.3. The optical chamber was left open to facilitate access with a glass microelectrode inserted through dura and used to record the cortical slow direct current (DC) potential with MultiClamp 200B amplifier at the site of imaged dendrites within layer I of somatosensory cortex. The Ag/AgCl pellet reference electrode (A-M Systems) was installed under the skin above the nasal bone. In a subset of mice, we assessed whether injection of a bolus of dibucaine hydrochloride into the tail vein (0.1 mL, 2.5 mg/kg, diluted in 0.9% NaCl) affects blood gas parameters. Mice were anesthetized with urethane (1.5 mg/g body weight) and one hundred microliters of blood were collected from a common carotid artery ∼1 h after dibucaine treatment and immediately quantified with a CG8+ blood gas cartridge and iStat1 blood gas analyzer (Abbott). Corresponding blood parameters were measured at ∼1 h in age- and sex-matched control mice that were anesthetized with urethane (1.5 mg/g body weight). Blood oxygen level and the acid–base balance were not statistically different in dibucaine-treated and control mice (t-test).

### 2PLSM

2PLSM images were collected with an IR-optimized 40×/0.8NA water immersion objective using the LSM 510 NLO META multiphoton system (Zeiss) mounted on the motorized upright Axioscope-2FS microscope. The scan module was directly coupled with the Spectra-Physics Ti:sapphire broadband mode-locked laser (Mai-Tai) tuned to 910 nm for 2-photon excitation. Three-dimensional time-lapse images were taken at 1 µm increments using 3× optical zoom, yielding a nominal spatial resolution of 6.86 pixels/µm (12 bits/pixel, 0.91 µs pixel time) across a 75×75 µm imaging field. Image Examiner (Zeiss) was used together with NIH ImageJ for image analysis. Dendritic beading was identified as the appearance of rounded regions extending beyond the diameter of the parent dendrite separated by “interbead” segments. Dendritic recovery was defined as the disappearance of rounded “beaded” regions.

### Photothrombotic Stroke Model

Photothrombotic occlusion was implemented as described recently [15]. A bolus of Rose Bengal was injected through the tail vein (0.03 mg/g, diluted to 10 mg/ml in 0.9% NaCl). With LSM 510 software, the perimeter (100 µm wide) of a square-shaped region of interest (ROI, 1270×1270 µm) was irradiated with the 514 nm laser line through a 10×/0.3NA water immersion objective. The central square (1070×1070 µm) was not illuminated by the laser. Photothrombosis was stopped immediately upon occurrence of initial induced spreading depolarization recorded in the vicinity of imaged dendrites approximately in the center of the ROI. A bolus of dibucaine hydrochloride was injected into the tail vein (0.1 mL, 2.5 mg/kg, diluted in 0.9% NaCl) immediately after the onset of photothrombotically-induced spreading depolarization (or at 30 min after the onset of induced spreading depolarization in a subset of experiments).

### Statistics

Statistica (StatSoft) was used to evaluate significant differences between conditions with two-way ANOVA. Two-tailed unpaired Student's *t* test, two-way repeated measures (RM) ANOVA, one-way ANOVA, one-way RM ANOVA and Chi-Square test were used when applicable. The significance criterion was p<0.05. Data are presented as mean±s.e.m.

## Results

**Figure 1:**
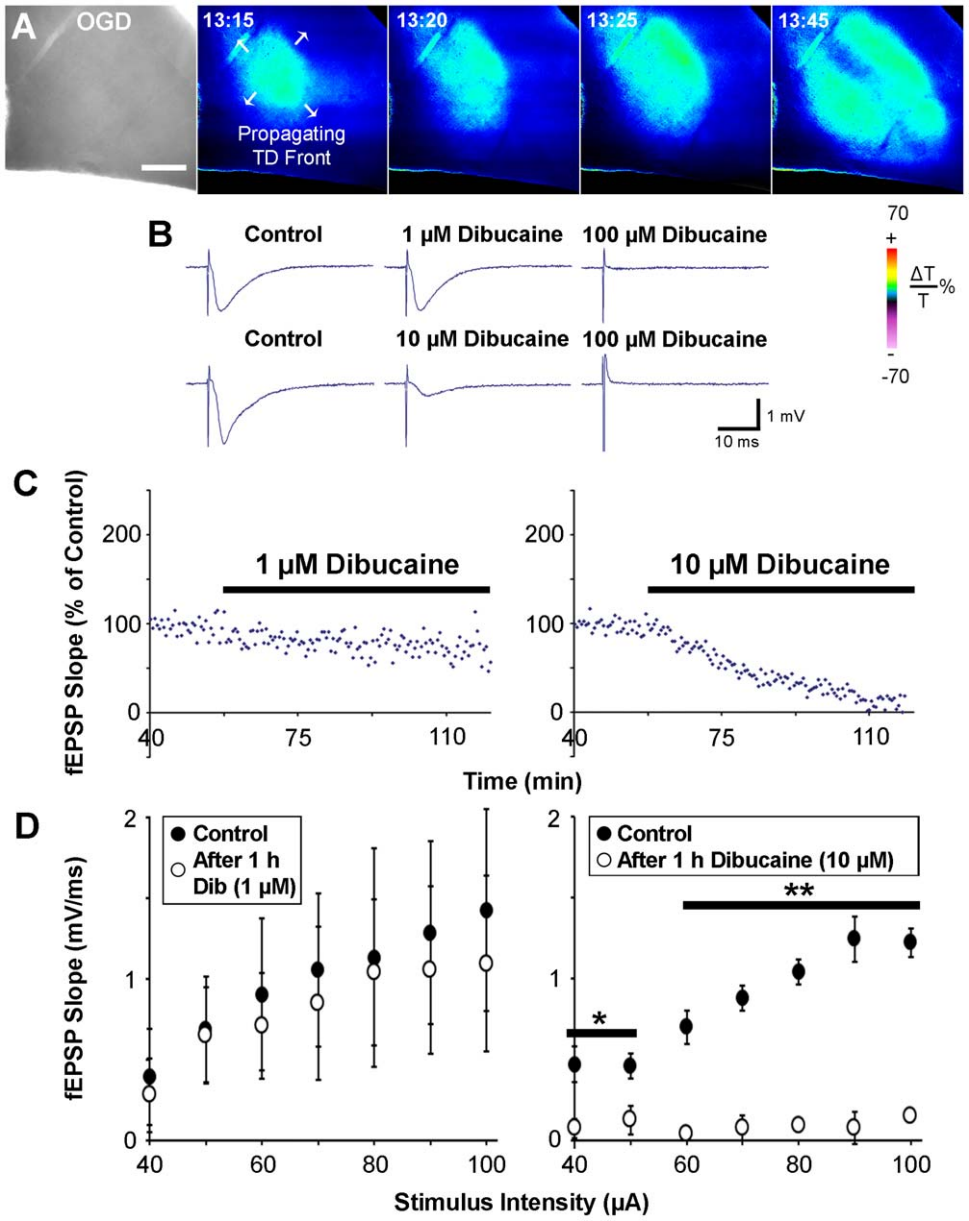
Global monitoring of terminal depolarization (TD) in human and mouse brain slices during dibucaine exposure. ***A***, A slice is transilluminated and transmitted light is collected by CCD camera to create the digital bright field image (left). Following OGD at 0 min, the gray matter displays a propagating wave of elevated LT (signifying terminal depolarization), represented by pseudocoloring according to changes in pixel values of the image (color scale: below, right). In this example, terminal depolarization is ignited within 13 minutes of OGD and spreads in all directions (arrows). Scale bar, 500 µm. ***B***, fEPSPs recorded from CA1 of *st. radiatum* in mouse hippocampal slices are not noticeably affected by 1 hour of superfusion with ACSF containing 1 µM dibucaine. 10 µM dibucaine partially inhibited the evoked synaptic response, while 100 µM dibucaine completely abolished synaptic activity. Each row represents traces recorded from a single slice. ***C***, Summary of synaptic responses during dibucaine exposure. Following 1 hour of control recording in standard ACSF, slices were superfused with ACSF containing 1 µM (left panel; n = 3 slices) or 10 µM dibucaine (right panel; n = 3 slices). At the end of 1 hour treatment with 1 µM dibucaine, ∼82% of the control fEPSP slope was preserved; 10 µM dibucaine reduced the fEPSP slope to ∼14% of control values (*p*<0.0001 for both; one-way ANOVA). ***D***, Input/output graphs showing mean fEPSP slope at various stimulus intensities before and after 1 hour treatment with either 1 µM (left) or 10 µM (right) dibucaine (n = 3 slices for each concentration; *p<0.05, **p<0.001; two-way ANOVA).

**Figure 2:**
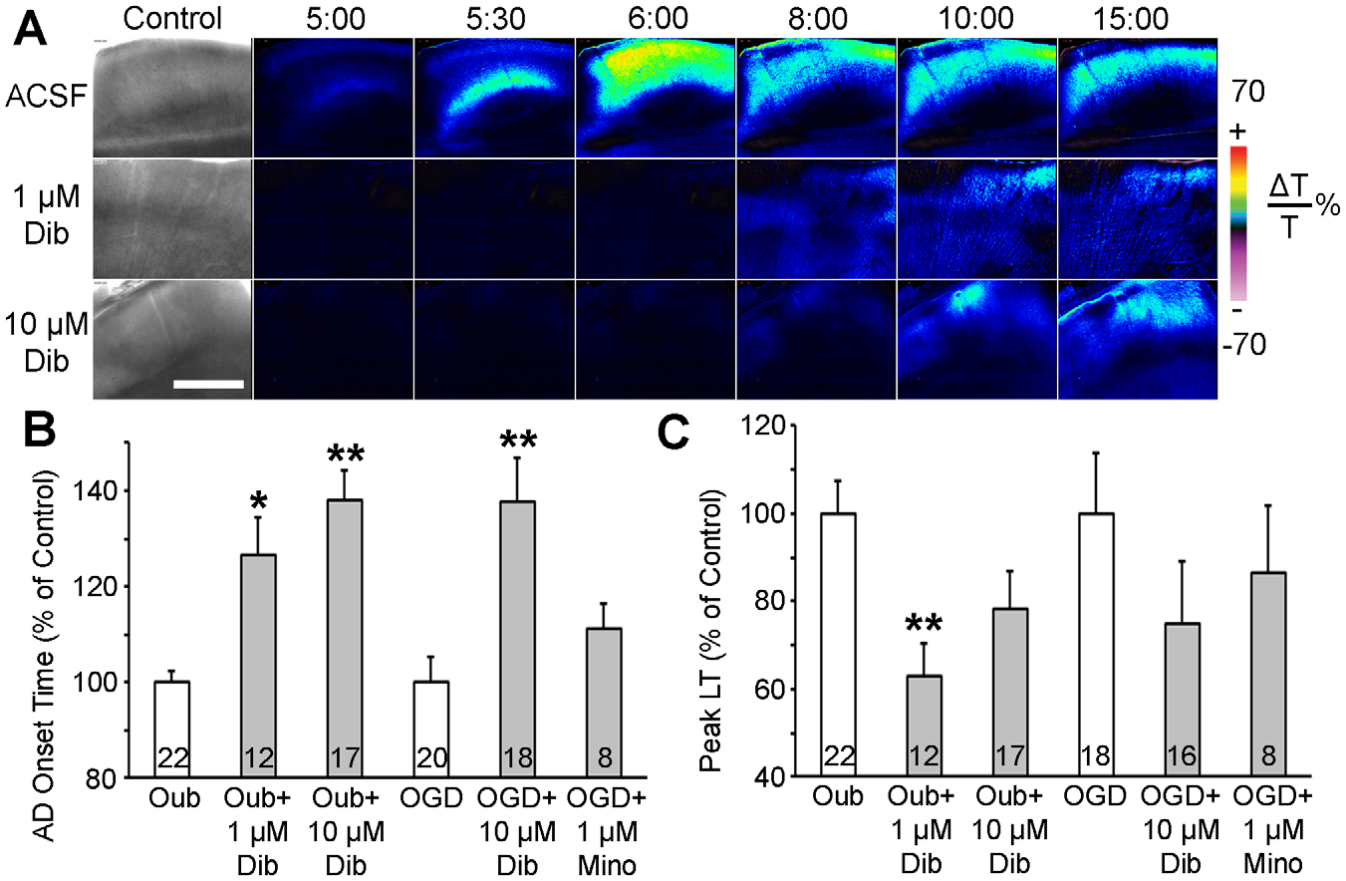
Dibucaine pretreatment delays terminal depolarization in human neocortical slices. ***A***, Changes in LT (ΔLT) are utilized to track terminal depolarization in real time. The first image in each row shows bright field image of the slice. A control untreated slice incubated in standard ACSF (top row) undergoes terminal depolarization within 5 minutes of superfusion with 100 µM ouabain. Pseudocolored elevated ΔLT indicates cell swelling. In slices pretreated with either 1 or 10 µM dibucaine for 60 min (middle and bottom rows, respectively), ouabain-induced terminal depolarization is delayed by 2.1 and 4.8 min respectively and has less impact in terms of cell swelling. All three slices were from the same patient (#7; see Patient Table S1). Scale bar, 1 mm. ***B***, Pretreatment for 1 hour with dibucaine significantly increases the latency to terminal depolarization onset induced by 100 µM ouabain or OGD when compared to untreated control slices from the same patient. ***C***, Peak cell swelling during terminal depolarization in the same slices pretreated with dibucaine. The neuroprotective anti-inflammatory drug minocycline had no effect on terminal depolarization. Numbers of slices in each condition are indicated within each bar. Values are shown as percent of control (untreated slices from the same patient). Asterisks indicate significant differences from control (*p<0.05, **p<0.001, two-way ANOVA).

To test candidate neuroprotectants' ability to mitigate terminal depolarization in human tissue, we first confirmed that propagating terminal depolarization can be reliably generated in human neocortical slices from pediatric patients. A near-complete breakdown of the ion concentration gradients associated with propagating terminal depolarization causes dramatic neuronal and astroglial swelling, dendritic beading and spine loss within seconds, leading to acute cell death [7], [27], [31]–[33]. Terminal depolarization-induced swelling of neurons and glia results in increased tissue transparency, appearing as an elevated LT wave [22], [27], [28], [34], [35]. Terminal depolarization rapidly induces dendritic beading which scatters light, lowering LT in swollen tissue [22], [27], [36]. By imaging changes in LT we verified that terminal depolarization can be evoked in human slices exposed to OGD (Fig. 1A) or 100 µM ouabain (Fig. 2A, top row). A wave of elevated LT, signifying terminal depolarization, was elicited between 6–17 min during OGD (10.91±0.56 min; n = 27 slices) and between 5–10 min during exposure to ouabain (6.83±0.26 min; n = 22 slices). Terminal depolarization spread throughout gray matter at 2.31±0.42 mm/min. Terminal depolarization onset and speed of propagation were independent of patients' age and gender, as well as the cortical region of the resected tissue. Thus, neocortical slices from human pediatric brain tissue were viable and supported terminal depolarization.

We next investigated whether dibucaine can inhibit terminal depolarization in human slices. In control slices, terminal depolarization was induced by superfusion with ACSF containing the Na^+^/K^+^-ATPase inhibitor ouabain (Fig. 2A, top row; Video S1). Slices from the same patient were pretreated for 1 hour with either 1 or 10 µM dibucaine-containing ACSF, then ouabain was co-superfused with dibucaine-containing ACSF to induce terminal depolarization (Fig. 2A, middle and bottom rows respectively; Videos S2, S3). These concentrations were tested in mouse hippocampal slices for effects on evoked synaptic activity (Fig. 1B–D). Similar to our recent study in rat neocortical slices, 1 µM dibucaine largely preserved synaptic function [22]. The images in Fig. 2A show a noticeably subdued response to terminal depolarization in dibucaine-pretreated human slices. Quantification shows increased latency to terminal depolarization onset (Fig. 2B) induced by ouabain in 1 and 10 µM dibucaine-pretreated slices. Due to the limited availability of human tissue only 10 µM dibucaine was tested against OGD-induced terminal depolarization. At this concentration dibucaine was effective in significantly delaying the onset of OGD-induced terminal depolarization (Fig. 2B). Decreased peak LT during ouabain-induced terminal depolarization was significant only in slices treated with 1 µM dibucaine (Fig. 2C). By comparison, pretreatment for 1 hour with the neuroprotective antibiotic minocycline (0.1,1, 5 and 100 µM) [33] had no effect on the cellular response to terminal depolarization. In addition, ANOVA has shown that tissue response to dibucaine treatment was independent of gender and age of these pediatric patients (data not shown). We confirm that human brain slices represent a viable approach for screening candidate therapeutic drugs that target spreading depolarization, and that dibucaine is one such drug.

**Figure 3:**
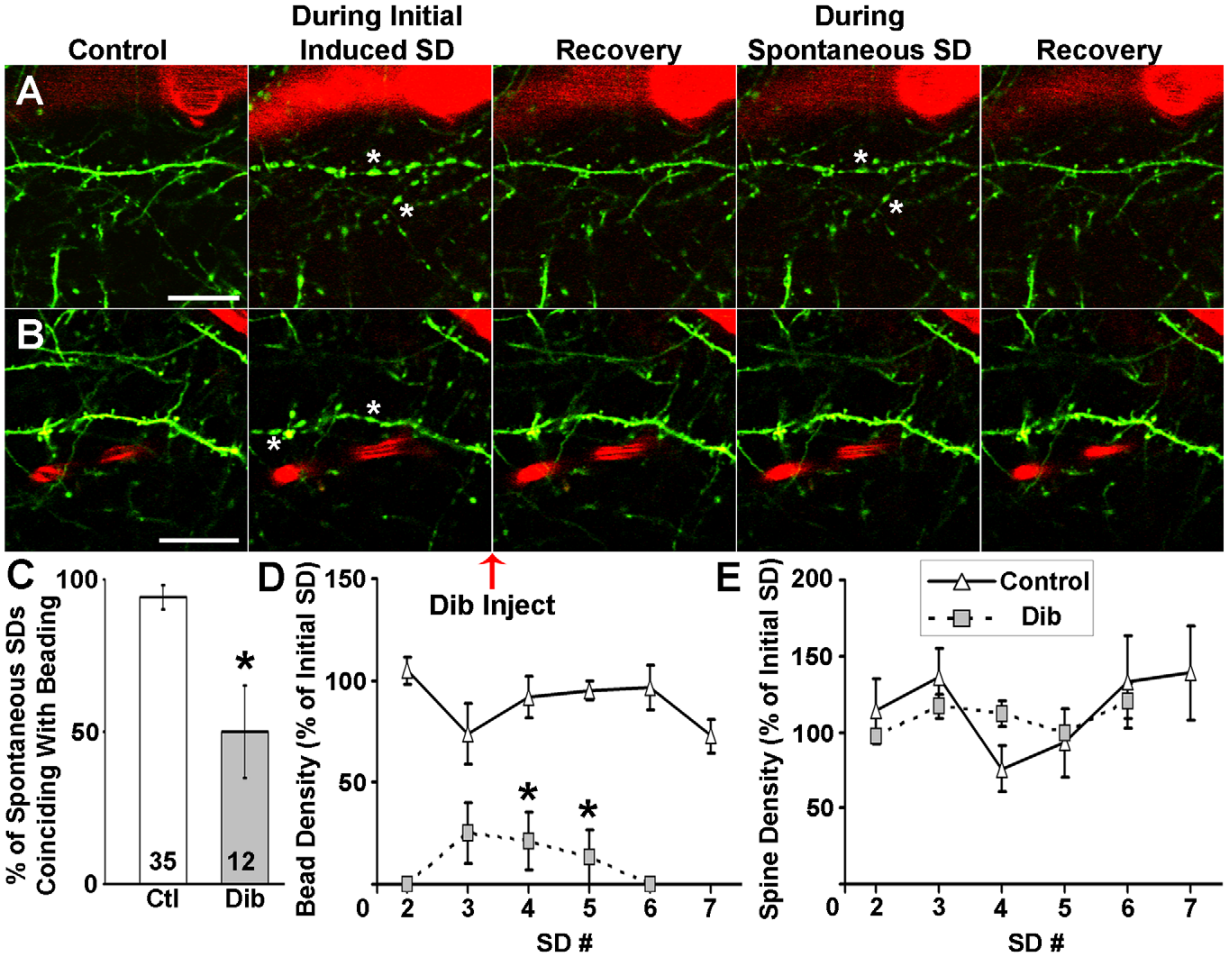
Dibucaine lessens dendritic beading induced by spontaneous spreading depolarizations (SDs) in penumbra. ***A***, *In vivo* 2PLSM image sequence of dendrites (green) and a nearby flowing blood vessel (red; blood flow is indicated by stripes within vessel) from intact mouse somatosensory cortex. The images were taken from the center of the penumbra-like “area at risk”, surrounded by partially occluded blood vessels [15]. The dendrites bead (asterisks) during the initial spreading depolarization induced by cortical photothrombosis. The dendrites rapidly (<3 min) recover but become beaded again by the next spontaneous spreading depolarization even in the presence of a flowing blood vessel before recovering once more. Scale bar, 20 µm. ***B***, Similar sequence to that shown in ***A***, except dibucaine (2.5 mg/kg) was injected into the tail vein immediately after the onset of induced spreading depolarization. As in ***A***, the dendrite rapidly recovers from beading (asterisks) induced by the initial spreading depolarization, but unlike the dendrites shown in ***A***, the dendrite in the dibucaine-treated animal does not bead during a spontaneous spreading depolarization. Scale bar, 20 µm. ***C***, Summary showing significantly decreased occurrence of dendritic beading (*p<0.05; Chi-Square Test) when dibucaine is administered i.v. immediately following onset of the initial spreading depolarization. ***D***, Bead density during spontaneous spreading depolarizations is significantly decreased with dibucaine while ***E***, spine density remains unaffected. Densities were assessed from random 30 µm segments of 3 dendrites in each imaging field as observed in maximum intensity projections of three-dimensional stacks in 5 control and 8 dibucaine treated mice. Data points (representing averages taken from individual dendrites) were only used when an image was taken near the peak of a recorded spreading depolarization. Values are shown as percentages of bead (***D***) or spine (***E***) density immediately after induced spreading depolarization (*p<0.05; two-way RM ANOVA).

After observing dibucaine's ability to delay the onset of terminal depolarization in human brain slices and temper its effects in rodent slices (Douglas et al., 2011), we then wanted to see if its protective properties would extend to the ischemic penumbra *in vivo* by preventing injury to fine synaptic circuitry caused by recurrent waves of spontaneous depolarizations. We recently used a photothrombotic occlusion model [37] which employs the photosensitive dye Rose Bengal (RB) to create a focal ischemic lesion enclosing a penumbra-like “area at risk” in intact mice [15]. We used 2PLSM in the center of this area at risk to show that acute neuronal injury represented by dendritic beading was highly correlated with the occurrence of the initial spreading depolarization induced during RB photoactivation as well as with subsequent spontaneous spreading depolarizations (Fig. 3A). Nearly all spontaneous spreading depolarizations correlated with dendritic beading regardless of whether there was a nearby flowing blood vessel. This beading was typically reversible in the presence of nearby flowing blood vessels [15]. Here, dibucaine was administered immediately after the onset of photothrombotically-induced spreading depolarization. Though dibucaine did not inhibit the occurrence of spontaneous spreading depolarizations (12 spontaneous spreading depolarizations from 8 animals, compared to 35 spontaneous spreading depolarizations in 24 control animals), dendritic structure remained remarkably stable following recovery from initial induced spreading depolarization (Fig. 3B). Dibucaine significantly reduced the percentage of spontaneous spreading depolarizations that were temporally correlated with dendritic beading (Fig. 3C). When beading did occur during spontaneous spreading depolarization, bead density was decreased (Fig. 3D) though spine density was unaffected (Fig. 3E). Dibucaine's prevention of dendritic beading did not appear to be related to local perfusion since the frequency of observations of flowing versus non-flowing vessels near imaged dendrites did not change with recurring spontaneous spreading depolarizations (p = 0.7; Chi-Square Test). Intriguingly, in a subset of 4 mice in which dibucaine injection was delayed until 30 min after photothrombotically-induced initial spreading depolarization, no beading was seen following dibucaine administration. Additionally, dibucaine treatment did not affect the quantitative features of spontaneous spreading depolarizations (p>0.05, Mann–Whitney U test, data not shown), such as amplitude (potential difference between start of SD and the lowest point of the DC deflection), duration (time between the points corresponding to half the DC amplitude and the same potential during recovery), maximum rate of depolarization (taken from steepest 4 s segment of SD), and onset time (relative to time of initial induced SD onset and time since the previous SD). Nevertheless, dibucaine-treated mice had significantly fewer incidents of terminal dendritic beading (i.e. beading without further recovery), occurring in just 1 of 11 mice (compared to 13 of 17 control mice).

## Discussion

We simulated ischemia in human neocortical slices to test the efficacy of candidate anti-stroke drugs in mitigating the effects of terminal depolarization. Ouabain-induced depolarization closely resembles terminal depolarization induced by OGD, and therefore both treatments are often used in rodent neuroprotective studies [21], [22], but rarely in human slices. Fundamental differences between rodent and human brain have long been considered as possible reasons why neuroprotective drugs that were effective in rodent models have failed in clinical stroke trials [23]. Energy failure during stroke is universal between species, but cerebral energy metabolism, blood flow and brain size vary. The human brain is larger and gyrated, reflecting increased size and number of neurons [7]. Phylogenetic changes are also reported, as human astrocytes are bigger, more complex and diverse [38]. Spreading depolarization under normoxic conditions can be elicited in human neocortical slices by potassium application, but the threshold is higher than in rat slices [39]. Human and rodent neocortical slices are different with respect to the latency of terminal depolarization onset induced by OGD (∼11 min human vs. ∼5.5 min rat slices [22]) or by ouabain (∼7 min human vs. ∼5 min rat slices [22]). Spreading depolarization cannot be triggered in the brains of newborn animals [7], but human neocortical slices obtained from even very young pediatric patents (∼2 y.o.) support terminal depolarization. We have therefore used an *in vitro* assay with human slices to test whether drugs shown to be effective in rodent models are also effective in human tissue.

Patient variables including age, gender, seizure etiology, resected cortical region, duration of the seizure disorder, age of onset, histopathological findings and history of medication may systematically affect tissue response. For those reasons, we always compared control and drug-treated experiments within tissue from the same patient and severely sclerotic tissue was excluded. The lack of “normal” tissue is a problem inherent to studies conducted on live human brain tissue *in vitro*, because obviously normal tissue cannot be removed. Nevertheless, testing candidate neuroprotectants in human brain slices was recognized in “The 2007 Feinberg lecture: a new road map for neuroprotection” as one crucial new step for developing novel therapies [24]. Epilepsy resections are the best source of differentiated viable human brain tissue available, and other investigators have used such tissue in similar studies [39], [40]. It is conceivable that the sensitivity of terminal depolarization to dibucaine in slices from the epileptic cortex may differ from normal cortex. “Non-epileptic” normal tissue is sometimes available, such as in the example shown in Fig. 2A where part of the occipital cortex was removed to access a benign tumor deeply located in the ventricle. This example is not different from our observations made in slices from epileptic cortex.

Terminal depolarization persistently resists pharmacological abrogation with blockers of voltage or ligand-gated ion channels [41], with the only successful attempts using a cocktail of glutamate receptor antagonists and voltage-dependent channel blockers [42], [43]. However, Yamada and co-authors [44] used rat hippocampal slices to show that 10 µM dibucaine prolonged the latency to OGD-induced terminal depolarization, decreased the maximal slope of this depolarization and induced partial recovery of neuronal membrane potential after reoxygenation/normoglycemia. Additionally, by measuring changes in LT and field potentials, Douglas and co-authors [22] reported that 1 µM dibucaine remarkably inhibits terminal depolarization in rat neocortical slices without altering synaptic function. Though dibucaine did not abolish terminal depolarization in human neocortical slices in our current studies, we observed that ouabain-induced terminal depolarization onset was delayed at the 1 µM concentration that preserved synaptic responses [22], indicating that this concentration could be well-tolerated therapeutically with minimal electrophysiological consequences. Terminal depolarization induced by OGD is more difficult to block than by ouabain [21], [22], perhaps because all energy requiring processes are inhibited by OGD, not just the Na^+^/K^+^-ATPase as in case of ouabain. OGD-induced terminal depolarization was delayed by 10 µM dibucaine in our experiments. Due to the limited availability of the human tissue, effectiveness of 1 µM dibucaine against OGD-induced terminal depolarizations was not tested in the current study, so future experiments (pending tissue availability) may be required to test lower concentrations of dibucaine against OGD-induced terminal depolarization. Furthermore, these findings from tissue resected from pediatric patients should ideally be compared to that from aged brains that are more at risk for stroke. Nevertheless our current study shows that dibucaine-like drugs could prove to be capable in protecting human brain from spreading depolarization-induced injury. It should be noted, though, that dibucaine is only approved for topical use by the FDA and may not be safe via an intravenous route. Its sister drug, lidocaine, is used to treat cardiac arrhythmias and its major side effects (e.g. CNS depression and convulsions) indicate that it crosses the blood brain barrier (BBB). Indeed, lidocaine was experimentally shown to be readily available for transport into the brain parenchyma across the BBB [45]. When lidocaine was injected i.v. in rabbits (4 mg/kg), it resulted in seizures with the cortical brain lidocaine level at 80±20 µg/ml and CSF lidocaine at 21±5 µg/ml [46]. Dibucaine injected i.v. can also cause seizures, as has been shown in animal models [47]. Additionally, accidental dibucaine overdose in pediatric patients results in seizures within minutes, indicating that it crosses the BBB [48].

Propagating terminal depolarization is difficult to spatially monitor in slices without complicated electrode arrays. Therefore, we used changes in LT to monitor this depolarization in time and space. In submerged slices, terminal depolarization-induced cellular swelling is the major source of increased LT [27], [28], [36]. Alternatively, direct imaging of dendritic injury and recovery with 2PLSM is a powerful approach to unambiguously monitor structural changes. Recently we have used 2PLSM imaging of single GFP-expressing neurons in mouse slices to demonstrate that dibucaine pretreatment was remarkably effective in reversing terminal depolarization-induced neuronal somata swelling and dendritic beading, with complete protection in some experiments [22]. Therefore, future 2PLSM experiments will be necessary to address the neuroprotective properties of dibucaine at the level of dendrites and dendritic spines in human slices.

Our study marks the first time that dibucaine-mediated protection of synaptic circuitry was confirmed in the penumbral zone *in vivo*. In the photothrombotic model, there is injury to the endothelium and microvasculature and early (within 15 minutes) breakdown of the BBB as evidenced by Evans blue leakage in the ring zone and some in the central penumbra [37]. Texas Red dextran (70 kDa) also leaks into the brain parenchyma after photothrombosis as assessed by signal detection outside capillaries [49], [50]. Perhaps the much lighter dibucaine (379.9 MW) can leak quickly under these conditions, thus providing protection for neurons and their dendrites. Because we administered dibucaine after the induction of photothrombosis, we were not able to assess the ability of dibucaine to delay the onset of the initial spreading depolarization as in our slice experiments. Instead, the critical findings of our *in vivo* experiments involved dibucaine's effect on spreading depolarization-induced dendritic beading. Terminal dendritic beading during ischemia is an early sign of acute injury leading to neuronal death [15], [51], [52]. Though the nonirradiated penumbra-like area at risk inside the lesion undergoes progressive hypoperfusion [15], penumbral dendrites have the ability to recover from spreading depolarization-induced beading, but this recovery is finite and depends on the presence of nearby flowing capillaries and thus the amount of energy available [15], [49], [50]. Remarkably, spreading depolarization-induced dendritic beading was significantly decreased following administration of dibucaine in intact mice subjected to focal photothrombotic occlusion. Importantly, terminal dendritic beading was observed only in one dibucaine-treated animal, suggesting that dibucaine-induced uncoupling of dendritic beading from the passage of recurrent spreading depolarizations protects dendrites by delaying terminal injury. Future studies using a different model where spreading depolarizations are frequently observed (e.g. middle cerebral artery occlusion) will be necessary to test whether infarct size is reduced by dibucaine.

The igniting mechanism of spreading depolarization during ischemia is of major interest, because drugs targeting spreading depolarization may protect the penumbra from progressively damaging depolarization waves [4], [21]. Sodium channel opening largely contributes to the depolarizing event although other channels are involved [7]. This may underlie the finding that sodium channel blockers are effective in delaying spreading depolarization but are not able to stop it once initiated [7], [53]. Thus, inhibition of terminal depolarization initiation by dibucaine likely involves an interaction with a sodium conductance because of its high binding affinity to the sodium channel [22]. Indeed, delaying the terminal depolarization onset should not be surprising for 10 µM dibucaine, because at this concentration dibucaine blocks enough sodium channels to inhibit action potentials and synaptic transmission. However, as revealed here and in our previous work [22] dibucaine inhibits terminal depolarization onset at 1 µM yet still preserves synaptic function. There are other reports where spreading depolarization was delayed by sodium channel blockers at concentrations that were too low to affect action potentials [54]–[56]. Remarkably, dibucaine (at 1 µM) displayed higher potency in inhibiting terminal depolarization onset in rodent slices than any other voltage-gated sodium channel blockers [22].

The mechanism by which dibucaine prevents dendritic beading despite the continued occurrence of spreading depolarizations is unclear. Spreading depolarization is characterized by severe disturbances of ion concentration gradients, resulting in the redistribution of ions across the membranes of metabolically compromised neurons [4], [7], [16]. Mammalian pyramidal neurons lack functional aquaporins, thus the molecular pathways by which they accumulate osmotically obligated water and swell during spreading depolarizations is uncertain [27]. Spreading depolarization possibly engages activation of a large conductance, such as pannexin-1 hemichannels [57], [58]. This should result in bulk water influx through the osmotically-tight neuronal compartment [27] leading to neuronal swelling and dendritic beading. It has been proposed that an interaction with such large ischemia-induced conductances would be neuroprotective [21] and may explain dibucaine's ability to prevent dendritic beading. Importantly, the success of experiments with dibucaine confirms that human brain slices are a suitable model system to identify and study potentially useful therapeutics targeting spreading depolarization. Furthermore, the protection of dendrites from the damaging effects of recurring spontaneous depolarizations is a surprising finding which suggests that a useful anti-stroke drug's therapeutic action may extend beyond mere inhibition of the spreading depolarization and that recording spreading depolarizations alone may not entirely reveal a drug's potential therapeutic benefit.

## Supporting Information

Video S1 Time series showing changes in LT evoked by 15 min of 100 µM ouabain exposure in a neocortical slice prepared from tissue obtained from a 7 y.o. female patient (patient # 7, see Patient Table S1). Time 0 corresponds to the beginning of ouabain exposure. A slice superfused with standard ACSF undergoes propagating terminal depolarization within ∼5 min of ouabain application. The terminal depolarization is imaged as an elevated LT (blue-yellow pseudocoloring signifying cell swelling).
(MOV)

Video S2 Similar time series to Video S1 of a slice from the same patient (patient #7) pretreated for 1 h with ACSF containing 1 µM dibucaine. Dibucaine incubation considerably delays the latency to the propagating terminal depolarization onset (6.8 min) and decreases peak cell swelling in the slice during co-superfusion with ACSF containing 100 µM ouabain and 1 µM dibucaine.
(MOV)

Video S3 Similar time series to Video S1 and S2 of a slice from the same patient (patient #7) pretreated for 1 h with ACSF containing 10 µM dibucaine. Dibucaine incubation considerably delays the latency to the propagating terminal depolarization onset (9.4 min) and decreases peak cell swelling in the slice during co-superfusion with ACSF containing 100 µM ouabain and 10 µM dibucaine.
(MOV)

Table S1 Patients in this study. Notes: FCD, focal cortical dysplasia [classification from: [59] Palmini A, Najm I, Avanzinin G et al. Terminology and classification of cortical dysplasias. Neurology 2004; 62 (suppl 3):S2–S8], DNT, Dysembryoplastic neuroepithelial tumour.
(DOCX)
