# Supplementary material for: Dibucaine Mitigates Spreading Depolarization in Human Neocortical Slices and Prevents Acute Dendritic Injury in the Ischemic Rodent Neocortex
Source: PLoS One. 2011 Jul 15;6(7):e22351. doi: 10.1371/journal.pone.0022351 (PMC3137632; doi:10.1371/journal.pone.0022351)
Supplement: Table S1 — Patients in this study. Notes: FCD, focal cortical dysplasia [classification from: [59] Palmini A, Najm I, Avanzinin G et al. Terminology and classification of cortical dysplasias. Neurology 2004; 62 (suppl 3):S2–S8], DNT, Dysembryoplastic neuroepithelial tumour. (DOCX) [file pone.0022351.s004.docx]

**Table S1**. Patients in This Study

| **PATIENT** | **GENDER** | **AGE** | **RESECTED CORTICAL REGION** | **PATHOLOGICAL FINDINGS** |
| --- | --- | --- | --- | --- |
| 1 | F | 11 | Parietal | FCD type 1A |
| 2 | M | 11 | Frontal | FCD type 1B |
| 3 | M | 3 | Frontal | FCD type 1B |
| 4 | M | 15 | Frontal | FCD type 2B |
| 5 | F | 10 | Frontal | FCD type 1B |
| 6 | F | 15 | Temporal | FCD type 1B |
| 7 | F | 7 | Occipital | Ventricle DNT (WHO grade I), No pathology in the occipital cortex |
| 8 | M | 3 | Parietal | FCD type 1B |
| 9 | F | 15 | Temporal | FCD type 1B |
| 10 | M | 13 | Frontal | DNT WHO grade I |
| 11 | M | 15 | Temporal | FCD type 1B |
| 12 | M | 4 | Frontal | Unknown |
| 13 | F | 2 | Parietal | FCD type 1B |
| 14 | F | 6 | Parietal | FCD type 1A |
| 15 | M | 16 | Temporal | Astrocytosis due to previous surgery |
| 16 | M | 10 | Frontal | FCD type 1A |
| 17 | M | 15 | Occipital | FCD type 2A |
| 18 | M | 3 | Temporal | Infiltrating astrocytoma (WHO grade II) |
| 19 | M | 10 | Frontal | FCD type 1A |
| 20 | M | 13 | Temporal | FCD type 1B |

Notes: FCD, focal cortical dysplasia [classification from: Palmini A, Najm I, Avanzinin G et al. Terminology and classification of cortical dysplasias. Neurology 2004; 62 (suppl 3):S2-S8], DNT, Dysembryoplastic neuroepithelial tumour
